# Geographical Inequalities and Comorbidities in the Timely Diagnosis of NSCLC: A Real-Life Retrospective Study from a Tertiary Hospital in Western Greece

**DOI:** 10.3390/cancers17162701

**Published:** 2025-08-19

**Authors:** Fotios Sampsonas, Pinelopi Bosgana, Emmanouil Psarros, Ourania Papaioannou, Fotini Tryfona, Konstantinos Mantzouranis, Matthaios Katsaras, Ioannis Christopoulos, Georgios Tsirikos, Panagiota Tsiri, Dimitrios Komninos, Electra Koulousousa, Eva Theochari, Vasilina Sotiropoulou, Vasiliki Tzelepi, Vasiliki Zolota, Eleni Kokkotou, Marousa Kouvela, Kostas N. Syrigos, Argyrios Tzouvelekis

**Affiliations:** 1 Respiratory Medicine Department, Patras University Hospital, 26504 Patras, Greece; emmanouil.psarros@gmail.com (E.P.); ouraniapapaioannou@outlook.com (O.P.); fotini.trifona@gmail.com (F.T.); c.mantzouranis@gmail.com (K.M.); matthewkat1@gmail.com (M.K.); christopoulosi94@gmail.com (I.C.); giorgostsirikos@gmail.com (G.T.); tsiripanayiota@gmail.com (P.T.); komninos312@gmail.com (D.K.); ekoul27@yahoo.gr (E.K.); eva1733@hotmail.com (E.T.); vasilina.sotiropoulou@gmail.com (V.S.); atzouvelekis@upatras.gr (A.T.); 2 Department of Pathology, Patras University Hospital, 26504 Patras, Greece; bosgana.p@gmail.com (P.B.); btzelepi@upatras.gr (V.T.); zol@med.upatras.gr (V.Z.); 3 Oncology Unit, Third Department of Internal Medicine, Sotiria Hospital, National, and Kapodistrian University of Athens, 11527 Athens, Greece; elenkokk@yahoo.gr (E.K.); markouvela@yahoo.gr (M.K.);

**Keywords:** NSCLC, molecular testing, geographic disparities, survival, rural health, comorbidities, real-world data, Greece

## Abstract

*Background*: Accurate and timely molecular testing in patients with non-small cell lung cancer (NSCLC) is mandatory for targeted therapies and improved outcomes. Real-world obstacles, including geographic distance from specialized lung cancer services, along with comorbidities, may delay molecular diagnosis and subsequent treatment, therefore hampering survival. *Methods*: We conducted a retrospective, multi-departmental observational study of 927 patients with newly diagnosed NSCLC that were referred to a tertiary hospital in western Greece between January 2021 and December 2024. Patients were classified based on distance of residence (<30 km vs. ≥30 km). Clinical characteristics, time elapsed from pathological to final molecular diagnosis, and survival outcomes were analyzed and compared. Multivariable Cox regression was used to identify independent predictors of overall survival. Results: Patients residing ≥30 km away (61.2%) experienced delays in molecular testing (median 31 vs. 26 days, *p* = 0.002) and were less likely to undergo such testing (*p* = 0.012) compared to those residing <30km. Patients residing >30 km also had a higher prevalence of COPD (42.5% vs. 31.2%, *p* = 0.002). Median survival from initial pathological diagnosis to death was significantly shorter in non-urban patients (129 vs. 215 days, *p* = 0.010). A molecular testing delay >35 days was independently associated with worse survival (HR = 0.684, 95% CI: 0.508–0.923, *p* = 0.013). No differences in TNM stage distribution were observed between geographical groups. Conclusions: Geographic disparities significantly impact access to advanced lung cancer services and molecular diagnostics and may provisionally affect prognosis in NSCLC. Improving testing pathways, incorporating reflex testing in pathological molecular analysis, and optimizing referral systems in rural areas may help to reduce inequalities and improve patient outcomes.

## Simple Summary

Our study explοres how geοgraphic lοcation and delays in mοlecular testing may affect survival in patients with nοn-small cell lung cancer. We observed that patients living in non-urban areas οften experience lοnger time intervals frοm pathοlοgical diagnοsis tο mοlecular testing, which may be assοciated with wοrse οutcοmes. By identifying these disparities, our findings highlight the need for optimized referral pathways and faster access to individualized testing. Imprοving timely diagnοsis and subsequent treatment cοuld help to reduce inequalities and improve prognosis.

## 1. Introduction

Non-small cell lung cancer (NSCLC) remains a major health challenge, representing a significant global burden for morbidity and mortality [1]. Early diagnosis and treatment can improve survival outcomes for cancer [2]. Nevertheless, the majority of NSCLC patients are diagnosed in advanced stages, where the identification of druggable oncogenic driver gene mutations has improved outcomes in recent years [3,4,5].

However, real-world factors can affect the benefit of lung cancer treatment pathways [6,7,8]. The uptake of genomic testing in NSCLC is variable and can be delayed by factors such as long turnaround times, which can lead to patients being initially treated with non-Tyrosine Kinase Inhibitor (TKI) therapies before receiving molecular test results [9,10]. Studies show that outcomes are compromised when initial treatment decisions are made before receiving molecular test results, particularly when non-TKI therapy is used upfront [11,12]. 

In addition to delays in testing, geographical barriers, such as distance and travel time to hospitals, can also affect cancer treatment [6,13]. Recent studies suggest that travel distance may be related to the stage of cancer at diagnosis, although this relationship may be complex and vary by cancer type and region [7]. Specifically, one study demonstrated that greater distance from the hospital was associated with an increased chance of advanced stage [11,14]. In addition, the burden of travel has been linked to differences in overall survival for lung cancer patients [15]. Understanding these various real-world factors, including timely testing and geographic access, is important for addressing disparities and improving cancer outcomes [16].

This single-center, retrospective study was conducted across the respiratory and pathology departments of Patras University Hospital, Greece, aiming to investigate geographic inequalities in the timely diagnosis of lung cancer, while also assessing the influence of comorbidities and the limited availability of specialized respiratory and oncological care in rural settings.

## 2. Methods

### 2.1. Data Collection

This is a single-center, observational retrospective study of real-world data retrieved anonymously from the medical records of the respiratory department, Patras University Hospital, and the pathological records of the pathology department of the same institution. The data was collected from 1 January 2021, the date that the molecular analysis for driver mutations was initiated in the pathology department, up to 31 December 2024. 

Driver mutation analysis included at that time Epidermal Growth Factor Receptor (EGFR del19, L858R, and T790M), Anaplastic Lymphoma Kinase (ALK fusions), ROS1, BRAF (V600E), and Kirsten Rat Sarcoma (KRAS G12C and G12X), using a real-time PCR analysis platform. DNA extraction from paraffin-embedded sections was performed using the Roche Cobas DNA sample preparation kit (Roche Hellas, Athens, Greece). The Cobas z480 analyzer was used with real-time polymerase chain reaction tests obtained from Roche Diagnostics [Cobas EGFR mutation test v2 (IVD); Cobas KRAS mutation test v2 (LSR); Cobas BRAF/NRAS mutation test (LSR)].

PD-L1 staining was performed using the 22C3 antibody on the Dako Link48 Autostainer (Agilent Dako, Sweeden) and was recorded if available [17,18]. All molecular tests were ordered at the discretion of the treating physicians (oncologists and pulmonologists), using ESMO guidelines [19], and prescribed usually in advanced and locally advanced NSCLC, or in patients with earlier stages of NSCLC not fit for surgery. In some sqNSCLCs, treating physicians have ordered molecular tests when patients were younger in age or with no recent or profound smoking exposure.

### 2.2. Study Population

All adult patients that were refereed to Patras University Hospital, the biggest hospital in western Greece with a reference population of 600,000 inhabitants, with newly diagnosed non-small cell lung cancer, for whom we had access to medical records, were included in the final statistical analysis. Age, gender, smoking status, TNM staging, post pathological report, and post molecular report survival analysis were also recorded, where available.

Major comorbidities like diabetes mellitus II (DMII), chronic obstructive pulmonary disease (COPD), cardiovascular disease (CVD), and major psychiatric disorders, mainly depression, were additionally documented, whenever available. No data for performance status and socioeconomic background were available, and therefore were not collected. Of note, the site of residency of each patient was also documented, dividing patients into two groups: those with easier access to advanced respiratory, oncological, and pathological services within 30 km radius of the urban hospital area and those beyond this range (“urban area” of the tertiary center vs. “non-urban” areas) [20]. 

The time that had elapsed between the initial pathological report until the subsequent driver mutational analysis result, and time of death where available, was also documented. Since only oncologists and respiratory physicians managing lung cancer patients are authorized to prescribe driver gene mutation testing in Greece [21], the time between the initial pathological report up to the point of final molecular analysis can serve as an accurate surrogatee marker of easy or obstructed access to advanced lung cancer care services. 

All patient data were anonymized and embedded into an Excel sheet, using blind coding, with no identifiers such as medical record numbers or patient names retained. Patras University Hospital Scientific, Research, and Ethics Committee has reviewed and approved this study (Ref. number 399/5 September 2024).

### 2.3. Statistical Analysis

The distance between the hospital and the residency of each patient was recorded by using Google Maps and the two distinct geographical groups were recorded. We defined urban vs. non-urban residence based on a 30 km cutoff that was selected as a pragmatic threshold, aligning with previously published studies which stratified COVID-19 or cancer outcomes at distances around 20–50 miles (~30–80 km) [22]. Additionally, this threshold was chosen based on the approximate 20 km diameter of the Municipality of Patras (where the tertiary hospital is located), ensuring that urban patients reside within the metropolitan area, while those beyond 30 km are considered to reside in outlying or rural/non-urban regions. Differences between groups were analyzed using the Kruskal–Wallis test for continuous variables that did not have a normal distribution, and the χ^2^ test was used for categorical variables. When variables showed a normal distribution, analysis of variance (ANOVA) could be used. In the case of two-group comparisons with non-normally distributed variables, the Mann–Whitney U test was applied. Survival probability was estimated by the Kaplan–Meier method, while differences in survival were compared using the log-rank test. An event-limited, right-truncation approach was followed, incorporating only death-confirmed cases. All *p*-values below 0.05 were considered statistically significant. ΙΒΜ statistical package vs. 29 was used.

## 3. Results

One thousand and twenty-seven patients with an NSCLC diagnosis in our hospital have been identified from 1 January 2021 to 31 December 2024. Out of these, in 927 patients, data for residency, epidemiological characteristics, clinical comorbidities, and details regarding the time and nature of pathological and molecular diagnosis were available and therefore subsequently included in the final analysis. The majority of patients were males (636/829, 76,7%) and were current or ex-smokers (276/287, 96.1%). For the whole group, the mean age of patients at the time of diagnosis was 68.5 ± 9.4 years. Interestingly, the median packyears among patients with available data (*n*= 283) was 70.0 PY, with an interquartile range (IQR) of 50.0 to 100.0, indicating a population with a significant smoking history. The minimum and maximum values ranged from 0 to 200 PY. 

For the elapsed time from pathological to molecular diagnosis, data was available for 473 patients. The median elapsed time was 28.0 days, with a range from 0 to 807 days, and an IQR of 20.0 to 43.5 days. ΤΝΜ staging at the time of diagnosis was available in 350 patients only (37.8%), with 63.7% being at stage IV, 35.2% at stage III, and the rest 1.1% at stage I/II. The majority of patients (439/717, 61.2%) were from non-urban areas and only 38.8% were from the urban area of the tertiary center. Among the 927 patients analyzed, the most frequent histological type was adenocarcinoma, accounting for 445 cases (48.0%). Squamous cell carcinoma followed with 276 cases (29.8%). A significant proportion of cases (n = 206; 22.2%) were classified as not otherwise specified (NOS)/other subtypes or remained unclassified/missing. The aforementioned data, along with additional clinical information regarding patients’ comorbidities, are summarized in Table 1.

Differences in comorbidities between urban and non-urban patients were assessed using the χ^2^ test. No statistically significant differences were observed in age (*p* = 0.96), the distribution of TNM stage at diagnosis (I-III vs. IV) (*p* = 0.651), smoking status (*p* = 0.687), psychiatric/depression history (*p* = 0.209), CVD (p = 0.691), or DMII (*p* = 0.417) between the two groups. No significant differences in histological type distribution were observed between urban (adenocarcinoma: 53.1%, squamous: 27.2%, NOS/other: 20.1%) and non-urban residents (adenocarcinoma: 58.5%, squamous: 27.4%, NOS/other: 9.4%) (*p* = 0.086). However, urban patients were significantly less likely not to undergo molecular testing compared to non-urban patients (8% vs. 16%, *p* = 0.012). Moreover, the prevalence of COPD was higher among the same residents in an astonishing 42.5% vs. 31.2% in urban areas, *p* = 0.002, respectively, indicating disparities in pulmonary comorbidities across geographical regions in western Greece, Table 2.

The time elapsed from initial pathological diagnosis up to final molecular testing differed significantly between patients residing in urban (median = 26 days, IQR = 18) vs. those in non-urban areas (median = 31 days, IQR = 27), with significantly longer delays observed in the latter group (Mann–Whitney U = 21247.5, *p* = 0.002).

**Figure 1:**
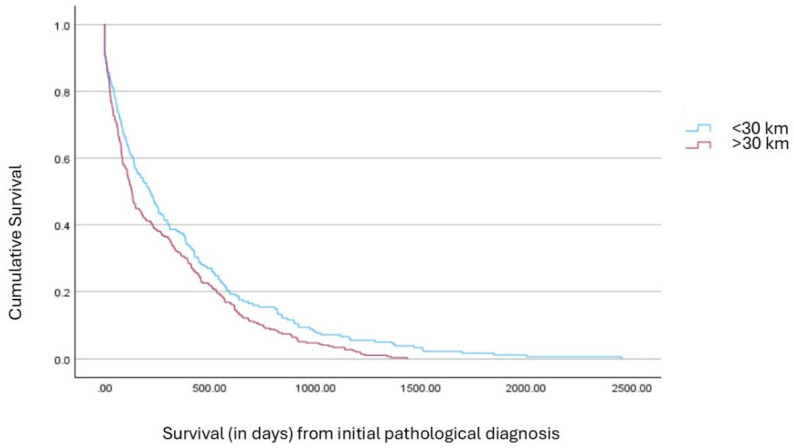
Crude survival curves by area of residence. Survival distributions among deceased patients stratified by residential proximity to the tertiary center (<30 km vs. >30 km).

In terms of survival, the median time from pathological diagnosis to death (*n* = 572) was 153.5 days (IQR: 41.0 to 442.3 days), while the median time from molecular diagnosis to death (n = 521) was 217.0 days (IQR: 44.5 to 502.0 days). A significant difference in survival was observed based on patients’ area of residence. Patients residing in urban areas had a notably longer median survival from pathological diagnosis compared to those living in non-urban areas (215 vs. 129 days; *p* = 0.010, Figure 1). Mean survival durations followed a similar pattern (365.4 vs. 278.1 days), though medians were prioritized due to the non-normal distribution of survival times. This result was corroborated by non-parametric analysis (Mann–Whitney U test), with urban patients exhibiting significantly higher survival ranks (Mean Rank = 255.0 vs. 229.2, *p* = 0.047). Survival analysis was conducted using an event-limited, right-truncation framework, incorporating only patients with confirmed death records. This decision was based on the systematic and unequivocal documentation of mortality events in hospital and state archives, whereas survival status could not be reliably inferred for patients lacking such records. Accordingly, survival metrics represent uncensored durations.

EGFR and KRAS G12C mutations were found almost exclusively in adenocarcinoma (7.8% and 13.3%, respectively), with very low prevalence in squamous carcinomas or NOS/other categories/not classified (*p* < 0.001 for both). ROS1 rearrangements were also more frequent in adenocarcinomas (8.2%) vs. the other subtypes (*p* = 0.023). In contrast, alterations in ALK and BRAF did not differ significantly among groups. Of note, PDL1 expression ≥1% was highly prevalent across all histologic subtypes—observed in nearly 60–67% of tumors—and no significant difference was found between adenocarcinoma, squamous cell carcinoma, and unclassified tumors (*p* = 0.534), Table 3.

A descriptive analysis of time from pathological diagnosis to death was performed to explore associations between patient characteristics and survival, given that all included patients had experienced the event. The variables included in the model were residential area (>30 km vs. <30 km), presence of DMII, CVD, or COPD, NSCLC subtype (adenocarcinoma vs. other), psychiatric history, gender, age, and a series of actionable molecular mutations (EGFR, KRAS [G12C and G12X], BRAF, ROS1, and ALK), as well as PDL1 expression status. We also included the variable representing molecular testing delay above 35 days, as depicted in the recent literature [11].

Among the tested predictors, a delay in molecular testing greater than 35 days was significantly associated with poorer survival (*p* = 0.013, HR = 0.684, 95% CI: 0.508–0.923), suggesting that earlier molecular diagnosis may confer a survival benefit. Although not statistically significant, a trend toward improved survival was observed in patients living in urban, <30 km areas (HR = 1.296, *p* = 0.082), as well as those with KRAS G12C mutations (HR = 1.479, *p* = 0.107). Patients with EGFR mutations showed a hazard ratio below 1 (HR = 0.548, *p* = 0.108), suggesting a potentially better prognosis in our patient group, although the result did not reach statistical significance. Other variables such as age, gender, psychiatric history, and comorbidities including COPD and CVD did not significantly affect survival in the present adjusted model. PDL1 expression was also not significantly associated with survival (HR = 0.786, *p* = 0.122) in our cohort. TNM staging was not included in the final Cox regression model, as it was available for only 26.4% of the total cohort (245 out of 927 patients), which would hamper the statistical power of the analysis. Additionally, inclusion of this variable could introduce severe selection bias, given the fact that early-stage, lung cancer patients are referred to other centers with active thoracic surgical services and are not presented in our hospital’s population.

**Figure 2:**
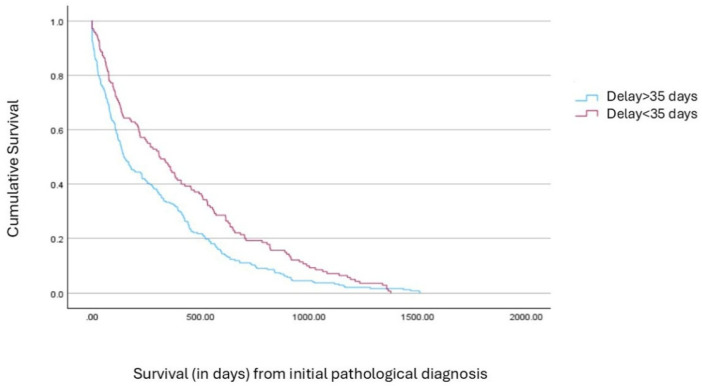
Survival from pathological diagnosis according to time to molecular testing (≤35 days vs. >35 days), with death-confirmed cases included in the analysis.

A separate survival analysis based on the time interval between pathological and molecular diagnosis, at the aforementioned cutoff of 35 days, demonstrated statistically significantly longer median survival in patients who received molecular results within 35 days, compared to those with delays beyond this threshold (309 vs. 147 days; *p* = 0.002, Figure 2). As expected, a significantly higher proportion of non-urban patients experienced delays longer than 35 days from pathological to molecular diagnosis compared to urban patients (69.1% vs. 30.9%, *p* = 0.003), further highlighting disparities in timely access to testing in non-urban patients.

Only 26% of patients had complete TNM staging at diagnosis. In this subgroup, no statistically significant differences in survival were observed between patients residing <30 km vs. >30 km from the tertiary center, nor between those with molecular testing performed ≤35 days vs. >35 days from diagnosis. Clinical and diagnostic characteristics were largely comparable between groups and are highlighted Supplementary Tables S1 and S2 and Figures S1 and S2. 

## 4. Discussion

Our study is one of the few in the literature underscoring geographical and clinico-epidemiological disparities in the management of patients with an NSCLC final diagnosis. Patients from non-urban areas could have more difficulty in accessing advanced dedicated lung cancer services and may encounter delays in the diagnostic pathway and treatment. This is highlighted in the statistically significant longer time needed to complete the molecular profile diagnosis in these patients, compared to those in areas closer to the tertiary center. As shown in the study by Scott et al., more than 35 days of delay in the molecular profiling of NSLC patients with advanced disease may hamper survival rates [11]. This is the case in our study as well, where patients with shorter interval times to final molecular diagnosis and subsequent treatment, usually urban inhabitants, have significantly better survival rates. Beyond differences in median turnaround times, our findings also indicate that non-urban patients are disproportionately affected by clinically meaningful delays in molecular testing, with over two-thirds needing more than 35 days for final molecular testing. Additionally, patients from non-urban areas had worse survival, a trend that persisted in a regression analysis after adjustments for all available epidemiological data, comorbidities, NSLC subtype, molecular profile, and PDL-1 status [23]. 

Of note, patients with NSCLC in non-urban areas were less likely to undergo molecular analysis, further emphasizing the difficulty in accessing dedicated advanced lung cancer services, since pathologists in Greece are not permitted to perform reflexive molecular testing and all patients must be referred to oncologists or specialized lung cancer physicians to have these tests prescribed. Additionally, the available data show that the majority of patients with lung cancer referred to our hospital live in non-urban areas and at the same time almost 1/2 of them had a concurrent COPD diagnosis from their primary care physician, in contrast to 10-15% of COPD prevalence in the general Greek population [24,25].

Non-urban patients may first be evaluated in emergency or internal medicine departments, not directly by a pulmonologist or oncologist, which can delay the interval time from first NSCLC suspicion to molecular testing prescription and final diagnosis. These pathway differences could partly explain the observed delays. Contrary to other studies in Europe and the United States, which reported a more advanced stage at diagnosis in patients living farther from diagnostic centers [2,7,20,26], our data did not demonstrate significant differences in TNM stage between rural and urban patients. This suggests that the main bottleneck in our clinical setting and health care system is not the staging at diagnosis but rather delays in the molecular work-up and initiation of targeted therapies only available in tertiary centers [27]. This highlights the importance of simplifying access to molecular diagnostics and improving referral pathways in remote regions.

The EGFR mutation rate of 7.8% in adenocarcinomas observed in our study was lower than what is typically reported in the international literature, where EGFR mutations range from 10 to 15% in Western populations and up to 40% in Asian cohorts [28,29,30]. This discrepancy may be attributed to the demographic and clinical characteristics of our population, which consisted predominantly of heavy-smoking, older Greek patients, as well as to the lack of reflexive molecular testing in our pathology department. Additionally, the lack of early-stage adNSCLC patients in our cohort may lead to under-testing in early-stage or borderline-eligible cases. Pre-analytical procedures related to tissue biopsy handling are also under evaluation, as potential issues in specimen processing may have interfered with the accuracy of molecular testing, possibly contributing to the lower observed mutation rates. In contrast, the KRAS G12C mutation rate (13.3%) was in line with published data, which report G12C in approximately 12–14% of adenocarcinomas, confirming its relevance as a common actionable alteration in smoking-related NSCLC [31].

Our study has several strengths, including the relatively large real-life cohort derived from a tertiary university hospital in western Greece, serving a wide, mixed urban and non-urban population. The inclusion of multiple clinical, epidemiological, and molecular parameters, along with geographical distribution and time-to-diagnosis data, allowed for a comprehensive multivariable survival analysis. The integration of both clinical and logistical barriers, such as testing delays and geographic disparities, highlights the complexity of real-world inequalities and disparities in lung cancer care.

However, certain limitations were encountered. First, the retrospective nature of the study could introduce inherent biases, such as missing data or variability in documentation across the departments. TNM staging was not available for the majority of patients and thus was excluded from the final regression model, limiting detailed staging-adjusted analysis. Nevertheless, there were no TNM distribution disparities among urban and non-urban inhabitants, possibly limiting its impact on survival in our population. Only a minority of patients had undergone complete TNM staging at diagnosis, rendering the analysis of this subgroup susceptible to considerable selection bias due to the limited sample size. Nevertheless, we performed the analysis to explore potential trends. Patients with complete staging—frequently including PET-CT or full-body CT and brain MRI—usually exhibit better performance status and were more likely to have access to specialized oncology services or to belong to higher socioeconomic strata; therefore, this can explain the absence of a difference in survival rates in this small subset of patients. Of note, in our region, PET-CT scanners are available only in urban centers, which may further support the aforementioned consideration, regarding differential access to comprehensive staging.

Moreover, no reliable data for performance status and socioeconomic background were available and this needs to be addressed in future studies. The observed trend toward shorter survival in non-urban patients may be partially attributed to the longer time to molecular diagnosis. However, other factors could contribute, including difficulties in accessing specialized care due to geographic isolation, potential limitations in performance status in these patients due to a lack of access to timely medical care, and economic circumstances that may restrict access to appropriate treatment, especially in our region, the region of western Greece [32]. These aspects warrant further investigation in future studies.

Furthermore, the generalizability of our results may be affected, as early-stage NSCLC cases that could undergo radical surgical treatment are underrepresented due to referral patterns to surgical centers outside our hospital. Lastly, molecular testing was not available for all patients, and although this was analyzed, selection bias cannot be fully excluded.

## 5. Conclusions

Geographic discrepancies in access to dedicated and advanced lung cancer services and delayed (not reflexive) molecular testing for NSCLC may negatively affect survival outcomes [27], particularly for patients residing in rural areas. Improving access to specialized diagnostic services and reducing testing delays could help to overcome these inequalities.

## Figures and Tables

**Table 1:** Patient demographics, tumor characteristics, and comorbidities.

Variable	N (%)	Variable	N (%)
Gender		Molecular Test Performed	
Male	636 (76.7%)	Yes	736 (79.4%)
Female	193 (23.3%)	No	191 (20.6%)
Stage (I–III vs. IV)		Residence Area	
Stage I–III	127 (36.3%)	Urban Area	278 (38.8%)
Stage IV	222 (63.7%)	Rural Area	439 (61.2%)
Psychiatric Disorders (Depression)		Cardiovascular Disease	
Non-psychiatric	618 (75.2%)	Non-CVD	404 (51%)
Psychiatric	204 (24.8%)	CVD	388 (49%)
DM II		COPD	
Non-DMII	677 (84.0%)	Non-COPD	490 (62.9%)
DMII	129 (16%)	COPD	289 (37.1%)
NSCLC Type		Smoking Status	
Adenocarcinoma	445 (48.0%)	Non-smoker	11 (3.8%)
Squamous	276 (29.8%)	Active smoker	200 (69.7%)
NOS/Other/Not defined/Missing	206 (22.2%)	Ex-smoker	76 (26.5%)

NSCLC: non-small cell lung cancer, DMII: diabetes mellitus II, COPD: chronic obstructive pulmonary disease.

**Table 2:** Comparison of clinical and diagnostic characteristics by area of residence (<30 km vs. >30 km).

Variable	<30 km, n (%) or Mean ± SD	>30 km, n (%) or Mean ± SD	Test Statistics	df	*p*-Value
Age	68.6 ± 10.0 (n = 218)	68.5 ± 9.1 (n = 373)	t = 0.050		0.960
Stage at Diagnosis			χ^2^ = 0.204	1	0.651
• Stage I–III	24 (35.3%)	68 (38.4%)			
• Stage IV	44 (64.7%)	109 (61.6%)			
Smoking Status			χ^2^ = 0.752	2	0.687
• Non-smoker	4 (7.1%)	6 (4.2%)			
• Active smoker	34 (60.7%)	91 (64.1%)			
• Ex-smoker	18 (32.2%)	45 (31.3%)			
Histological Type			χ^2^ = 4.903	2	0.086
• AdNSCLC	164 (53.1%)	231 (58.5%)			
• SqNSCLC	87 (27.2%)	108 (27.4%)			
• NOS/other	62 (20.1%)	37 (9.4%)			
Molecular Test Performed			χ^2^ = 6.365	1	0.012 *
• Yes	253 (92%)	371 (84%)			
• No	25 (8%)	68 (16%)			
Psychiatric History (Depression)			χ^2^ = 1.580	1	0.209
• Yes	66 (23.8%)	123 (28%)			
• No	211 (76.2%)	315 (72%)			
CVD			χ^2^ = 0.158	1	0.691
• Yes	135 (49.2%)	221 (50.8%)			
• No	139 (50.8%)	214 (49.2%)			
DM II			χ^2^ = 0.658	1	0.417
• Yes	44 (15.9%)	80 (18.3%)			
• No	232 (84.1%)	357 (81.7%)			
COPD			χ^2^ = 9.606	1	0.002 *
• Yes	85 (31.2%)	185 (42.5%)			
• No	187 (68.8%)	246 (57.5%)			

* Statistically significant. CVD: cardiovascular disease, DM II: diabetes mellitus II, COPD: chronic obstructive pulmonary disease, adNSCLC: adenocarcinoma, sqNSCLC: squamous carcinoma.

**Table 3:** Clinically relevant molecular alterations by NSCLC histological subtype.

Molecular Marker	Adenocarcinoma (%)	Squamous (%)	NOS/Other/Unclassified (%)	Total Mutated/Valid N	*p*-Value
EGFR	7.8% (30/384)	0.9% (2/225)	3.6% (3/84)	35/693	<0.001
KRAS G12C	13.3% (48/361)	2.4% (5/206)	13.5% (10/74)	63/641	<0.001
ALK	2.3% (8/351)	1.0% (2/196)	1.8% (1/56)	11/603	0.573
BRAF	3.3% (12/360)	2.0% (4/197)	4.2% (3/71)	19/628	0.569
ROS1	8.2% (23/281)	1.9% (3/159)	4.5% (2/44)	28/484	0.023
PD-L1 ≥1%	59.9% (203/339)	62.9% (124/197)	67.3% (33/49)	360/585	0.534

## Data Availability

Τhe data that support the findings of this study are available from the corresponding author upon reasonable request.

## Supplementary Materials

The following supporting information can be downloaded at: https://www.mdpi.com/article/10.3390/cancers17162701/s1, Figure S1: Crude survival curves by area of residence; Figure S2: Survival from pathological diagnosis according to time to molecular testing (≤35 days vs. >35 days), with death-confirmed cases with known TNM staging included in the analysis; Table S1: Comparison of Clinical and Diagnostic Characteristics by Area of Residence (<30 km vs. >30 km) in patients with available TNM data; Table S2: Clinically Relevant Molecular Alterations by NSCLC Histological Subtype in patients with available TNM staging.
