# Supplementary material for: Geographical Inequalities and Comorbidities in the Timely Diagnosis of NSCLC: A Real-Life Retrospective Study from a Tertiary Hospital in Western Greece
Source: Cancers (Basel). 2025 Aug 19;17(16):2701. doi: 10.3390/cancers17162701 (PMC12384472; doi:10.3390/cancers17162701)
Supplement: Supplementary file 1 [file cancers-17-02701-s001.zip › cancers-3811992-supplementary.pdf]

Supplementary Materials

Geographical Inequalities and Comorbidities in the Timely Diagnosis of NSCLC: A Real-Life Retrospective Study from a Tertiary Hospital in Western Greece

Supplementary Analysis

Among patients with available TNM staging (26.2% of the total cohort), no statistically significant differences were observed in overall survival between those residing <30 km and >30 km from the tertiary center (Figure S1). Similarly, survival did not differ between patients with molecular testing performed ≤35 days versus >35 days from pathological diagnosis (Figure S2). Baseline characteristics by distance from the center are shown in Table S1, with no major discrepancies between rural and non-rural areas. Distribution of clinically relevant molecular alterations by histological subtype is presented in Table S2.

Table S1. Comparison of Clinical and Diagnostic Characteristics by Area of Residence (<30 km vs. >30 km) in patients with available TNM data.

Variable	<30 km, n (%) or Mean ± SD	>30 km, n (%) or Mean ± SD	Test Statistics	df	*p*-Value
**Age**	69± 36.0 (n = 63)	68.7 ± 9.2 (n = 373)	t = 0.427		0.670
**Stage at Diagnosis**			χ^2 = 0.204	1	0.651
• Stage I–III	24 (35.3%)	68 (38.4%)			
• Stage IV	44 (64.7%)	109 (61.6%)			
**Smoking Status**			χ^2 = 0.752	2	0.687
• Non-smoker	4 (7.1%)	6 (4.2%)			
• Active smoker	34 (60.7%)	91 (64.1%)			
• Ex-smoker	18 (32.2%)	45 (31.3%)			
**Histological Type**			χ^2 = 4.179	2	0.124
• AdNSCLC	43 (64.1%)	91 (52%)			
• SqNSCLC	17 (25.4%)	69 (39.4%)			
• NOS/other	7 (10.5%)	15 (8.6%)			
**Molecular Test Performed**			χ^2 = 0.057	1	0.811
• Yes	43 (92%)	109 (84%)			
• No	25 (8%)	68 (16%)			
**Psychiatric History (Depression)**			χ^2 = 1.249	1	0.264
• Yes	18 (26.5%)	60 (33.9%)			
• No	50 (73.5%)	117 (66.1%)			
**CVD**			χ^2 = 0.032	1	0.858
• Yes	37 (55.2%)	100 (56.5%)			
• No	30 (44.8%)	77 (43.5%)			
**DM II**			χ^2 = 1.038	1	0.308
• Yes	14 (20.6%)	48 (27.3%)			
• No	53 (79.4%)	128 (72.7%)			
**COPD**			χ^2 = 1.008	1	0.315
• Yes	30 (44.7%)	92 (49.2%)			
• No	37 (55.3%)	85 (50.8%)			

CVD: Cardiovascular Disease, DMII: Diabetes mellitus II, COPD: Chronic obstructive Pulmonary Disease, adNSCLC: Adenocarcinoma, sqNSCLC: Squamous carcinoma.

Table S2. Clinically Relevant Molecular Alterations by NSCLC Histological Subtype in patients with available TNM staging.

Molecular Marker	Adenocarcinoma (%)	Squamous (%)	NOS/Other/Unclassified (%)	Total Mutated / Valid N	*p*-Value
EGFR	12.2% (10/82)	0% (0/39)	0% (0/15)	8 /141	0.072
KRAS G12C	12.2% (10/82)	5.3% (2/38)	0% (0/14)	12 /134	0.216
ALK	3.7% (3/81)	0% (0/34)	7.7% (1/13)	4 /128	0.353
BRAF	4.9% (4/82)	0% (0/36)	0% (0/14)	4 /142	0.284
ROS1	8.3% (5/60)	0% (0/30)	1% (1/11)	6 /101	0.259
PD-L1 ≥1%	60.2% (53/85)	58.5% (24/41)	66.7% (10/15)	360 /585	0.0.858

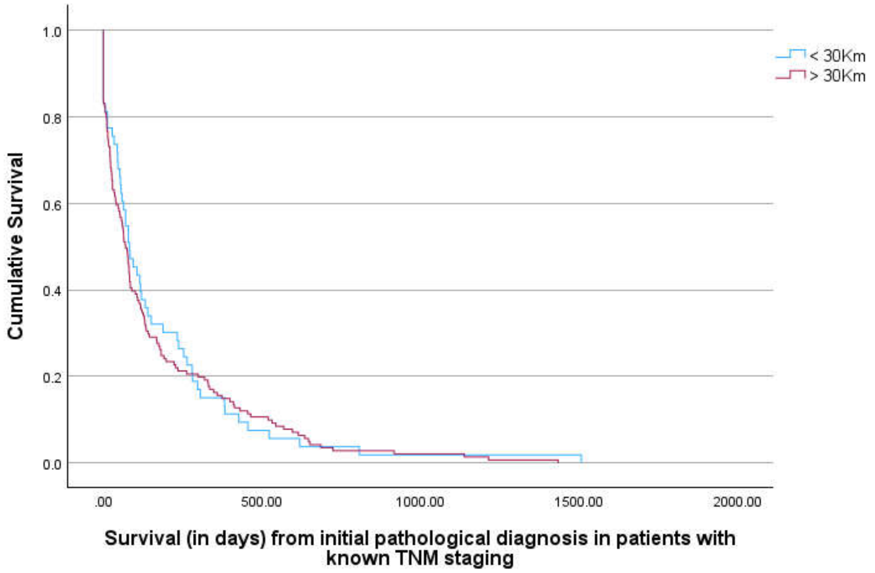

Figure S1. Crude survival curves by area of residence. Survival distributions among deceased patients with known TNM staging stratified by residential proximity to the tertiary center (<30 km vs. >30 km).

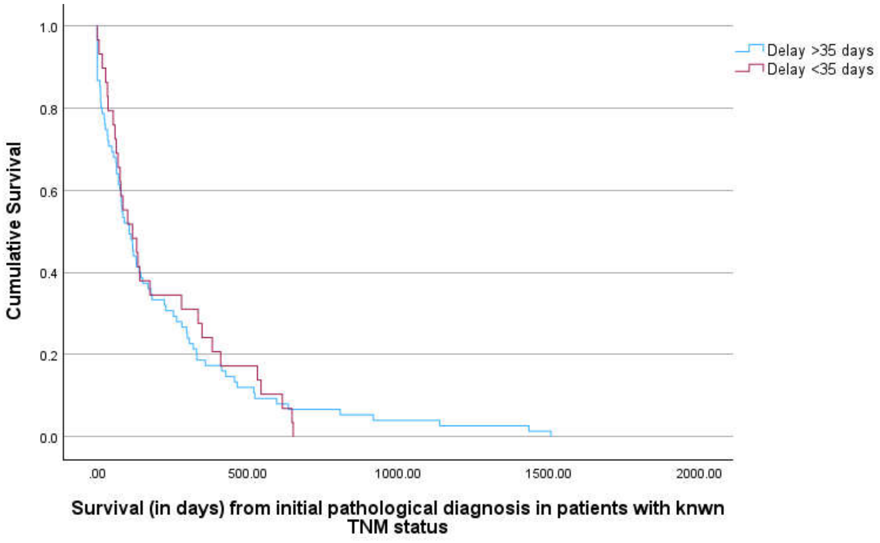

Figure S2. Survival from pathological diagnosis according to time to molecular testing (≤35 days vs. >35 days), with death-confirmed cases with known TNM staging included in the analysis.
